# Supplementary material for: The Small RNA Universe of Capitella teleta
Source: Front Mol Biosci. 2022 Feb 25;9:802814. doi: 10.3389/fmolb.2022.802814 (PMC8915122; doi:10.3389/fmolb.2022.802814)
Supplement: Supplementary file 1 [file DataSheet1.ZIP › Supplement/known/CAPTEscaffold_777_28950.pdf]

| | |
|---|---|
| Provisional ID | : CAPTEscaffold_777_28950 |
| Score total | : 303215.4 |
| Score for star read(s) | : 3.9 |
| Score for read counts | : 303205.4 |
| Score for mfe | : 1.5 |
| Score for randfold | : 1.6 |
| Score for cons. seed | : 3 |
| Total read count | : 594736 |
| Mature read count | : 594425 |
| Loop read count | : 0 |
| Star read count | : 311 |

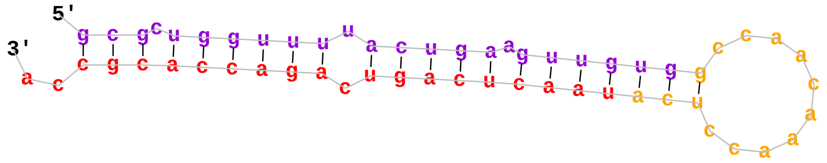


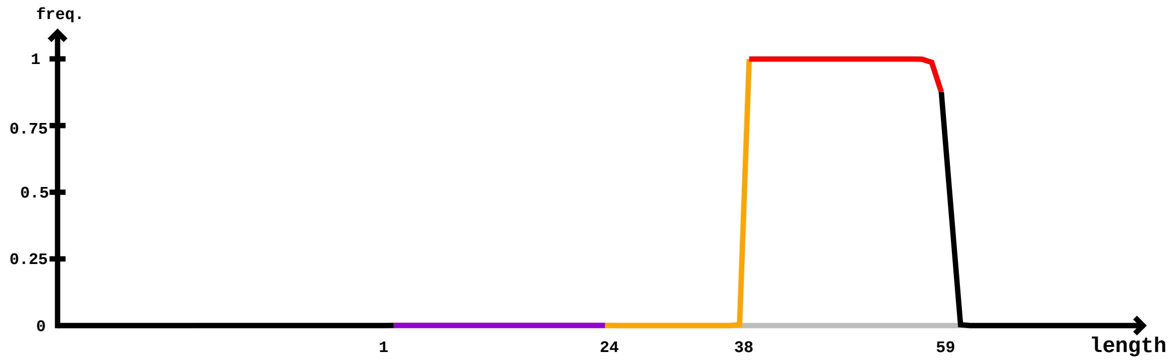


Star Mature

```
5'- cugucguguuauauuauuaucggagucaggauggcgcugguuuuacugaaguugug gccaacaaaccucauaacucagucagaccacgccauacugcucacuucaaacugc  -3'  obs
    cugucguguuauauuauuaucggagucaggauggcgcugguuuuacugaaguuguggccaacaaaccucauaacucagucagaccacgccauacugcucacuucaaacugc       exp
    cugucguguuauauuauuaucggagucaggauggcgcugguuuuacugaaguuguggccaacaaaccucauaacucagucagaccacgccauacugcucacuucaaacugc       known
    ......................(((.(((..(((((.((((((.(((((.(((((((..........)))))))))))).)))))))))))..))))))............      reads   mm   sample

    .............uuauuaucggagucaggaug..............................................................................      36      0    seq
    ..............uauuaucggagucaggaug..............................................................................      6       0    seq
    ...............auuaucggagucaggaug..............................................................................      2       0    seq
    ...............................uggcgcugguuuuacugaaguugu........................................................      1       0    seq
    .................................gcgcugguuuuacugaaguu..........................................................      6       0    seq
    .................................gcgcugguuuuacugaaguugu........................................................      23      0    seq
    .................................gcgcugguuuuacugaaguuguA.......................................................      3       1    seq
    .................................Ucgcugguuuuacugaaguugug.......................................................      2       1    seq
    .................................gcgcugguuuuacugaaguugug.......................................................      271     0    seq
    .................................gcgcugguuuuacugaaguuguggg.....................................................      2       0    seq
    .................................gcgcugguuuuacugaaguugugA......................................................      1       1    seq
    .................................gcgcugguuuuacugaaguuguggc.....................................................      2       0    seq
    .................................gcgcugguuuuacugaaguuguggccaaca................................................      1       0    seq
    ...................................................................ucauaacucagucagaccacgcc.....................      2       0    seq
    ....................................................................Aauaacucagucagaccacgcca....................      1       1    seq
    .....................................................................auaacucagucagaccacg.......................      2       0    seq
    .....................................................................auaacucagucagaccacgc......................      8       0    seq
    .....................................................................auaacucagucagaccacgcc.....................      182     0    seq
    .....................................................................Cuaacucagucagaccacgcc.....................      14      1    seq
    .....................................................................auaacucagucagaccacgccU....................      344     1    seq
    .....................................................................auaacucagucagaccGcgcca....................      1       1    seq
    .....................................................................auaacucGgucagaccacgcca....................      1       1    seq
    .....................................................................auaacucagucagaccaAgcca....................      1       1    seq
    .....................................................................auaacucagucagacAacgcca....................      5       1    seq
    .....................................................................auaacucagucagaccacgcca....................      976     0    seq
    .....................................................................Cuaacucagucagaccacgcca....................      233     1    seq
    .....................................................................auaacucagucagaccacgccG....................      3       1    seq
    .....................................................................Uuaacucagucagaccacgcca....................      2       1    seq
    .....................................................................aAaacucagucagaccacgcca....................      1       1    seq
    .....................................................................auaacNcagucagaccacgcca....................      1       1    seq
    .....................................................................auaGcucagucagaccacgcca....................      1       1    seq
```

Star Mature

```
cugucguguuauauuauuaucggagucaggauggcgcugguuuuacugaaguugugggccaacaaaccucauaacucagucagaccacgccauacugcucacuucaaacugc
.....................................................................auaaAucagucagaccacgcca....................	1	1	seq
.....................................................................auaacucagAcagaccacgcca....................	2	1	seq
.....................................................................auaacucagucagaccacgccau...................	16	0	seq
.....................................................................auaacucagucagaccacgccaA...................	14	1	seq
.....................................................................auaacucagucagaccacgccauU..................	3	1	seq
......................................................................uaacucaguAagaccacg.......................	1	1	seq
......................................................................uaacucagucagaccacU.......................	2	1	seq
......................................................................uaacucagucagaccaUg.......................	1	1	seq
......................................................................uaacucagucaAaccacg.......................	1	1	seq
......................................................................Aaacucagucagaccacg.......................	5	1	seq
......................................................................uaacucagucagaccGcg.......................	1	1	seq
......................................................................uaacucagucagaccacg.......................	414	0	seq
......................................................................uaacucaguUagaccacg.......................	1	1	seq
......................................................................uaacucagAcagaccacgc......................	1	1	seq
......................................................................uGacucagucagaccacgc......................	1	1	seq
......................................................................uaacucagucagaccacAc......................	1	1	seq
......................................................................uaacucagucagaccacgA......................	11	1	seq
......................................................................uaacAcagucagaccacgc......................	8	1	seq
......................................................................uaacuGagucagaccacgc......................	1	1	seq
......................................................................Gaacucagucagaccacgc......................	2	1	seq
......................................................................Naacucagucagaccacgc......................	2	1	seq
......................................................................uaacucagucagaccacgG......................	1	1	seq
......................................................................uaacucagCcagaccacgc......................	1	1	seq
......................................................................uaacucagucGgaccacgc......................	1	1	seq
......................................................................uaacucaguAagaccacgc......................	5	1	seq
......................................................................uaacucagucagaccacgU......................	38	1	seq
......................................................................Aaacucagucagaccacgc......................	41	1	seq
......................................................................uaacucagucagaccacgc......................	6778	0	seq
......................................................................uaacucagucCgaccacgc......................	1	1	seq
......................................................................uaacucGgucagaccacgc......................	2	1	seq
......................................................................uaacucagucagacUacgc......................	3	1	seq
......................................................................uNacucagucagaccacgc......................	1	1	seq
......................................................................uaaAucagucagaccacgc......................	1	1	seq
......................................................................uaacucaguGagaccacgc......................	1	1	seq
......................................................................uaacuAagucagaccacgc......................	5	1	seq
......................................................................uaacucagucagaUcacgc......................	2	1	seq
......................................................................uaacucagucagaccacCc......................	1	1	seq
......................................................................uaacucagucagaccaUgc......................	1	1	seq
......................................................................uaacucagucagacAacgc......................	4	1	seq
......................................................................uaacucaCucagaccacgc......................	2	1	seq
......................................................................uaacucagucagaccGcgc......................	1	1	seq
......................................................................uaacucaUucagaccacgc......................	2	1	seq
......................................................................uaacucagucagaccacUc......................	1	1	seq
......................................................................uaacucagucagaUcacgcc.....................	8	1	seq
......................................................................uaacuUagucagaccacgcc.....................	22	1	seq
......................................................................uaacucagucagaccacgcU.....................	118	1	seq
......................................................................uaacucGgucagaccacgcc.....................	15	1	seq
......................................................................uGacucagucagaccacgcc.....................	17	1	seq
......................................................................uaacucagucagaccacgcG.....................	5	1	seq
......................................................................uaacucagucaUaccacgcc.....................	5	1	seq
......................................................................uaacucaAucagaccacgcc.....................	6	1	seq
......................................................................uaacucagAcagaccacgcc.....................	14	1	seq
......................................................................uaGcucagucagaccacgcc.....................	3	1	seq
......................................................................uaacucagucagacUacgcc.....................	30	1	seq
......................................................................uaacucagucagaccacgUc.....................	6	1	seq
......................................................................uaacucagucaAaccacgcc.....................	13	1	seq
......................................................................uaacCcagucagaccacgcc.....................	3	1	seq
......................................................................uaacucagucagaccacAcc.....................	11	1	seq
......................................................................uaNcucagucagaccacgcc.....................	1	1	seq
......................................................................uaacucaguUagaccacgcc.....................	13	1	seq
......................................................................uaacucagucCgaccacgcc.....................	1	1	seq
......................................................................uaacucagucagaGcacgcc.....................	3	1	seq
......................................................................Naacucagucagaccacgcc.....................	17	1	seq
......................................................................uaacucagucagaAcacgcc.....................	6	1	seq
......................................................................uaacucagucagacGacgcc.....................	5	1	seq
......................................................................uaacGcagucagaccacgcc.....................	4	1	seq
......................................................................uaacucaUucagaccacgcc.....................	1	1	seq
......................................................................uaacucagucGgaccacgcc.....................	18	1	seq
......................................................................uaacucagucagaccacgGc.....................	3	1	seq
......................................................................uaacucagucagaccacgcA.....................	65	1	seq
```

```
                                Star                                 Mature
cugucguguuauauuauuaucggagucaggauggcgcugguuuuacugaaguuguggccaacaaaccucauaacucagucagaccacgccauacugcucacuucaaacugc
......................................................................uaacucagucagaccaNgcc.....................   1       1   seq
......................................................................uaacucagucagaccUcgcc.....................   5       1   seq
......................................................................uaacucagucagaccacgcc.....................   64711   0   seq
......................................................................Gaacucagucagaccacgcc.....................   35      1   seq
......................................................................uaacAcagucagaccacgcc.....................   64      1   seq
......................................................................uaaAucagucagaccacgcc.....................   9       1   seq
......................................................................uaacucagucagaccacUcc.....................   3       1   seq
......................................................................uaacucaguGagaccacgcc.....................   2       1   seq
......................................................................uaaGucagucagaccacgcc.....................   1       1   seq
......................................................................uaacucagGcagaccacgcc.....................   1       1   seq
......................................................................uaacucagucagaccaUgcc.....................   12      1   seq
......................................................................uaacucaguAagaccacgcc.....................   37      1   seq
......................................................................uaacucagucagaccGcgcc.....................   5       1   seq
......................................................................uaacucCgucagaccacgcc.....................   3       1   seq
......................................................................uaacuAagucagaccacgcc.....................   52      1   seq
......................................................................uaacucagucagaccacgAc.....................   15      1   seq
......................................................................uaacucaCucagaccacgcc.....................   6       1   seq
......................................................................uaaUucagucagaccacgcc.....................   3       1   seq
......................................................................uaacucagucagGccacgcc.....................   9       1   seq
......................................................................uaacuGagucagaccacgcc.....................   2       1   seq
......................................................................Caacucagucagaccacgcc.....................   9       1   seq
......................................................................uaacucUgucagaccacgcc.....................   6       1   seq
......................................................................uaacucagucUgaccacgcc.....................   3       1   seq
......................................................................uaUcucagucagaccacgcc.....................   5       1   seq
......................................................................uaacucagucagacAacgcc.....................   133     1   seq
......................................................................uaacuNagucagaccacgcc.....................   1       1   seq
......................................................................Aaacucagucagaccacgcc.....................   347     1   seq
......................................................................uaacucagucagUccacgcc.....................   9       1   seq
......................................................................uaacucagucaCaccacgcc.....................   2       1   seq
......................................................................uNacucagucagaccacgcc.....................   3       1   seq
......................................................................uaacucagucagaccaGgcc.....................   2       1   seq
......................................................................uaacucagCcagaccacgcc.....................   7       1   seq
......................................................................uaacucagucagaccaAgcc.....................   9       1   seq
......................................................................uaacucagucagaccacCcc.....................   2       1   seq
......................................................................uaacucagucagacAacgcca....................   944     1   seq
......................................................................uaacucagucaCaccacgcca....................   26      1   seq
......................................................................uaacucaAucagaccacgcca....................   44      1   seq
......................................................................uaacucagucagaccacUcca....................   44      1   seq
......................................................................uaacucagucaAaccacgcca....................   60      1   seq
......................................................................uGacucagucagaccacgcca....................   83      1   seq
......................................................................uaacucagucagCccacgcca....................   15      1   seq
......................................................................uaaUucagucagaccacgcca....................   24      1   seq
......................................................................uaaGucagucagaccacgcca....................   20      1   seq
......................................................................uaacucagucagUccacgcca....................   37      1   seq
......................................................................uaacucUgucagaccacgcca....................   34      1   seq
......................................................................uaacucaguAagaccacgcca....................   226     1   seq
......................................................................uaacucagucagaUcacgcca....................   51      1   seq
......................................................................uaacucagucagaccCcgcca....................   14      1   seq
......................................................................uaacucaguGagaccacgcca....................   20      1   seq
......................................................................uaacucaUucagaccacgcca....................   26      1   seq
......................................................................uaacucaCucagaccacgcca....................   60      1   seq
......................................................................uaCcucagucagaccacgcca....................   7       1   seq
......................................................................uaacucagucagaAacgcca.....................   72      1   seq
......................................................................uaacucCgucagaccacgcca....................   9       1   seq
......................................................................uaacucagucagaccacgUca....................   57      1   seq
......................................................................uaacucagucagacUacgcca....................   176     1   seq
......................................................................Naacucagucagaccacgcca....................   123     1   seq
......................................................................uaacucagucagaccacgcAa....................   281     1   seq
......................................................................uaacucagucNgaccacgcca....................   1       1   seq
......................................................................uaacucagucagaccacgccG....................   1373    1   seq
......................................................................Caacucagucagaccacgcca....................   45      1   seq
......................................................................uUacucagucagaccacgcca....................   4       1   seq
......................................................................uaacucagucagaccacgNca....................   1       1   seq
......................................................................uaacucaguUagaccacgcca....................   89      1   seq
......................................................................uaacCcagucagaccacgcca....................   48      1   seq
......................................................................Aaacucagucagaccacgcca....................   2162    1   seq
......................................................................uaacucagGcagaccacgcca....................   16      1   seq
......................................................................uaacuGagucagaccacgcca....................   18      1   seq
......................................................................uaacuNagucagaccacgcca....................   7       1   seq
......................................................................uaacucagucagaccaAgcca....................   89      1   seq
```

**Star** **Mature**

```
cugucguguuauauuauuaucggagucaggauggcgcugguuuuacugaaguuguggccaacaaaccucauaacucagucagaccacgccauacugcucacuucaaacugc
......................................................................uaacucagucagaccacAcca....................   80      1   seq
......................................................................uaacucagucagaccUcgcca....................   23      1   seq
......................................................................uCacucagucagaccacgcca....................   3       1   seq
......................................................................uaacucagucagaccacCcca....................   26      1   seq
......................................................................Gaacucagucagaccacgcca....................   247     1   seq
......................................................................uaacucagucagaccGcgcca....................   74      1   seq
......................................................................uaacucagucGgaccacgcca....................   106     1   seq
......................................................................uaacucagucagaccacgcGa....................   27      1   seq
......................................................................uaacucagucUgaccacgcca....................   16      1   seq
......................................................................uaacucagucCgaccacgcca....................   3       1   seq
......................................................................uaacucagucagaNcacgcca....................   1       1   seq
......................................................................uaUcucagucagaccacgcca....................   48      1   seq
......................................................................uaacucagucaUaccacgcca....................   35      1   seq
......................................................................uaacucagucagaccaGgcca....................   18      1   seq
......................................................................uNacucagucagaccacgcca....................   13      1   seq
......................................................................uaacucagAcagaccacgcca....................   180     1   seq
......................................................................uaacucagucagaccaUgcca....................   55      1   seq
......................................................................uaaAucagucagaccacgcca....................   53      1   seq
......................................................................uaacGcagucagaccacgcca....................   18      1   seq
......................................................................uaacucagucagaccacgcNa....................   1       1   seq
......................................................................uaacucagucagaccacgcUa....................   163     1   seq
......................................................................uaacuUagucagaccacgcca....................   201     1   seq
......................................................................uaacucagucagacGacgcca....................   28      1   seq
......................................................................uaNcucagucagaccacgcca....................   2       1   seq
......................................................................uaacucagucagGccacgcca....................   64      1   seq
......................................................................uaacucagucagaccacgcca....................   449297  0   seq
......................................................................uaacucagucagaccacgAca....................   99      1   seq
......................................................................uaacucagucagaGcacgcca....................   19      1   seq
......................................................................uaacucGgucagaccacgcca....................   135     1   seq
......................................................................uaacucagucagaccacgccN....................   2       1   seq
......................................................................uaacucagucagaccacgccU....................   48034   1   seq
......................................................................uaacucagucagaccacgGca....................   18      1   seq
......................................................................uaacucagucagaccacgccC....................   33      1   seq
......................................................................uaacAcagucagaccacgcca....................   396     1   seq
......................................................................uaacucagCcagaccacgcca....................   46      1   seq
......................................................................uaacuAagucagaccacgcca....................   309     1   seq
......................................................................uaGcucagucagaccacgcca....................   67      1   seq
......................................................................uaacucaguNagaccacgcca....................   1       1   seq
......................................................................uaacucaguAagaccacgccau...................   2       1   seq
......................................................................uaacucagucagaccacgcAau...................   6       1   seq
......................................................................uaacucagAcagaccacgccau...................   1       1   seq
......................................................................uaacucagucagacUacgccau...................   1       1   seq
......................................................................uaacucagucagaccacgccUu...................   79      1   seq
......................................................................uaacucUgucagaccacgccau...................   1       1   seq
......................................................................uaacucagucagaccacgccGu...................   2       1   seq
......................................................................uaacucagucagaccacgccau...................   5239    0   seq
......................................................................uaacucaguUagaccacgccau...................   3       1   seq
......................................................................uaacucagCcagaccacgccau...................   2       1   seq
......................................................................uaUcucagucagaccacgccau...................   2       1   seq
......................................................................Aaacucagucagaccacgccau...................   19      1   seq
......................................................................uaacuGagucagaccacgccau...................   1       1   seq
......................................................................uaacucaAucagaccacgccau...................   1       1   seq
......................................................................uaacucagucGgaccacgccau...................   2       1   seq
......................................................................uaacucGgucagaccacgccau...................   1       1   seq
......................................................................uaacucaUucagaccacgccau...................   2       1   seq
......................................................................uaacuAagucagaccacgccau...................   7       1   seq
......................................................................uaacuUagucagaccacgccau...................   3       1   seq
......................................................................uaacucagucagacAacgccau...................   16      1   seq
......................................................................uaacAcagucagaccacgccau...................   2       1   seq
......................................................................uaaUucagucagaccacgccau...................   1       1   seq
......................................................................uaacucagucagaccacgccaC...................   11      1   seq
......................................................................uaacucagucagaccacgccaG...................   290     1   seq
......................................................................uaacucagucagaccUcgccau...................   1       1   seq
......................................................................Gaacucagucagaccacgccau...................   3       1   seq
......................................................................uaacucagucagaccacgcUau...................   1       1   seq
......................................................................uaacucagucagGccacgccau...................   2       1   seq
......................................................................uaaAucagucagaccacgccau...................   1       1   seq
......................................................................uaacucagucagaccaUgccau...................   2       1   seq
......................................................................uaacucagucagaccacgccaA...................   5449    1   seq
......................................................................uaGcucagucagaccacgccau...................   1       1   seq
```

```
                                 Star                                 Mature
cugucguguuauauuauuaucggagucaggauggcgcugguuuuacugaaguuguggccaacaaaccucauaacucagucagaccacgccauacugcucacuucaaacugc
......................................................................uaacucagucagaccacgUcau...................    2     1    seq
......................................................................uaacucagucagaAcacgccau...................    1     1    seq
......................................................................uaacucagucagaccacgccauU..................    1058  1    seq
......................................................................uaacucagCcagaccacgccaua..................    1     1    seq
......................................................................uaacucagucagaccacgccaCa..................    2     1    seq
......................................................................uaacucagucagaccacgccaGa..................    5     1    seq
......................................................................Aaacucagucagaccacgccaua..................    1     1    seq
......................................................................uaacucagucagaccacgccauC..................    62    1    seq
......................................................................uaacucagucagaccacgccaAa..................    613   1    seq
......................................................................uaacucagucagaccacgccauG..................    7     1    seq
......................................................................uaacucagucagaccCcgccaua..................    1     1    seq
......................................................................uaacucagucagaccacgccaua..................    96    0    seq
......................................................................uaacucagucagaccacgccUua..................    3     1    seq
......................................................................uaacucagucagaccacgccauaU.................    8     1    seq
......................................................................uaacucagucagaccacgccaAac.................    4     1    seq
.......................................................................Uacucagucagaccacgc......................    1     1    seq
.......................................................................aacucagucagaccacgcc.....................    11    0    seq
.......................................................................Uacucagucagaccacgcc.....................    3     1    seq
.......................................................................Uacucagucagaccacgcca....................    29    1    seq
.......................................................................aacucagucagaccacgccU....................    2     1    seq
.......................................................................aacucagucagaccacgcca....................    20    0    seq
.......................................................................aacucagucagacUacgcca....................    1     1    seq
.......................................................................Uacucagucagaccacgccau...................    1     1    seq
.......................................................................aacucagucagaccacgccaA...................    1     1    seq
........................................................................acucagucagaccacgcca....................    5     0    seq
........................................................................acAcagucagaccacgcca....................    1     1    seq
...........................................................................................uacugcucacuucaaacugc    7     0    seq
```
